# Antibody seropositivity and endemicity of chikungunya and Zika viruses in Nigeria

**DOI:** 10.1186/s44149-023-00070-2

**Published:** 2023-03-23

**Authors:** Peter Asaga Mac, Philomena E. Airiohuodion, Raman Velayudhan, Shaistha Zubair, Markos Tadele, Jude, O. Aighobahi, Chukwuma Anyaike, Axel Kroeger, Marcus Panning

**Affiliations:** 1 Institute of Virology, University Medical Freiburg, Hermann Herder Str, 11, 79104 Freiburg, Germany; 2 World Health Organization, Special Programme for Research and Training in Tropical Diseases (TDR), Avenue Appia 20, 1211 Geneva 27, Switzerland; 3 World Health Organization, WHO/NTD Unit, Avenue Appia 20, 1211 Geneva 27, Switzerland; 4 Maldives National University, Buruzu, Magu, Male, Maldives; 5 Ethiopian Institute Of Agricultural Research/EIAR, Addis Ababa, Ethiopia; 6 Icon Clinical Research, Heinrich-Hertz Starsse 26, 63225 Langen Hessen, Berlin, Germany; 7 Federal Ministry of Health, National Tuberculosis and Leprosy ControlProgramme, Abuja, Nigeria; 8 Centre for Medicine and Society, Faculty of Medicine, University of Freiburg, Freiburg, Germany

**Keywords:** Chikungunya, Zika, Cocirculation, Seroprevalence, Nigeria, Endemicity, Malaria

## Abstract

Mosquito-borne infections are of global health concern because of their rapid spread and upsurge, which creates a risk for coinfections. chikungunya virus (CHIKV), an arbovirus disease transmitted by *Aedes aegypti* or *A. albopictus*, and malaria, a parasitic disease transmitted by *Anopheles gambiae*, are prevalent in Nigeria and neighbouring countries, but their burden and possible coinfections are poorly understood. In this study, we investigated the antibody seropositivity and endemicity of chikungunya and Zika viruses (ZIKV) in three regions of Nigeria. A cross-sectional sero-survey was conducted on 871 participants. Samples were collected from outpatients by simple random sampling. Analyses of the samples were performed using recomLine Tropical Fever for the presence of antibody serological marker IgG immunoblot with CHIKV VLP (virus like particle), ZIKV NS1 and ZIKV Equad according to manufacturers’ instructions and malaria RDT for malaria parasite. There was a significantly higher antibody seropositivity against CHIKV in the central region than in the northern and southern regions (69.5%, 291/419), while ZIKV-seropositivity (22.4%, 34/152) and CHIKV-ZIKV co-circulating antibody seropositivity (17.8%, 27/152) were notably higher in the southern region than in the central and northern regions. This investigation revealed an unexpectedly high antibody seropositivity and concealed endemicity of CHIKV and ZIKV in three Nigerian regions. The seropositivity of detectable antibodies differed among the three geographical locations.

**Supplementary Information:**

The online version contains supplementary material available at 10.1186/s44149-023-00070-2.

## Introduction

The rapid and continuous emergence of arthropod-borne viruses (arboviruses) poses a serious threat to public health. Local outbreaks are fuelled by various factors, such as urbanization, increased travel, and climate change (Asaga Mac et al. 2022; Ekong et al. 2022; Masika et al. 2022; Norman et al. 2020; Ali et al. 2022; Carrillo-Hernández et al. 2018). In malaria and dengue-endemic regions, the emergence of chikungunya virus (CHIKV) and Zika virus (ZIKV) creates intriguing and potentially alarming scenarios and could possibly be misdiagnosed as a malaria infection (Masika et al. 2022; Norman et al. 2020; Ali et al. 2022; Carrillo-Hernández et al. 2018; Olawoyin and Kribs 2020; Otu et al. 2019). In urban settings, such as Lagos in Nigeria and several other cities and regions in sub-Saharan Africa, all three viruses infect humans, with mosquitoes (primarily *Aedes aegypt*i and *Aedes albopictus*) as the major vector. They share common biological, ecological and economic factors (Olawoyin and Kribs 2020; Otu et al. 2019), leading to epidemiological synergy. Concurrent infections with two or more viruses are commonly reported (Norman et al. 2020; Abdullahi et al. 2020; Zambrano et al. 2016; Omatola et al. 2020; Iovine et al. 2017; Carrillo et al.  2018; Coelho et al. 2016; Oluwole et al. 2022; Perkins et al. 2016; Weaver and Lecuit 2015; Fauci and Morens 2016; Gardner et al. 2018; Bisanzio et al. 2018; Furuya-Kanamori et al. 2016; Messina et al. 2016). Zika virus can cause microcephaly and other birth defects during pregnancy (Paniz-Mondolfi et al. 2016; Rico-Mendoza et al. 2019; Joseph et al. 2021; Edwards et al. 2016; Diallo et al. 2018), and the long-term effects of chikungunya-induced chronic arthritis and associated cognitive disorders have been described (Ali et al. 2022; Rico-Mendoza et al. 2019; Joseph et al. 2021; Edwards et al. 2016; Diallo et al. 2018). A mutation (A226 V) in the E1 glycoprotein, which enhances CHIKV transmission, is one of the factors contributing to the global spread of the virus by *A. albopictus* (Rothan et al. 2018). In Nigeria, it can be very complicated to accurately diagnose arboviruses in healthcare facilities because there are limited staff with the necessary skills and molecular diagnostic tools to differentiate between the two arboviral infections. There are several serodiagnostic tests for arboviral infections, including the enzyme-linked immunosorbent assay (ELISA), neutralization test (NT), immunofluorescence assay (IFA), and hemagglutination inhibition test. Dengue and Zika infections can be reliably and specifically serologically diagnosed using PRNT (Asaga Mac et al. 2022; Olawoyin and Kribs 2020). However, PRNT is time consuming and requires a biosafety level 3 facility to handle live viruses. Comparatively, ELISA is simple and safe, but is hindered by cross-reactivity among flaviviruses (Asaga Mac et al. 2022; Ekong et al. 2022; Norman et al. 2020; Ali et al. 2022; Iovine et al. 2017; Carrillo et al. 2018). The potential outcomes of coinfection in vulnerable groups, such as pregnant and immunocompromised individuals, could lead to prolonged viremia and poor foetomaternal outcomes (Masika et al. 2022; Norman et al. 2020; Joseph et al. 2021; Edwards et al. 2016; Diallo et al. 2018; Zambrano et al. 2016). The process and consequences of coinfections are poorly understood in Nigeria and other African countries. Many imperative questions remain unanswered: Does coinfection with chikungunya virus/Zika virus alter the course of human diseases in Nigeria?

CHIKV and ZIKV have emerged as highly significant threats to public health in Nigeria and worldwide. Surveillance activity for arboviral infections in Nigeria is non-functional. The present study assessed antibody seropositivity, endemicity and varied spread of CHIKV, ZIKV and CHIKV-ZIKV antibody cocirculation in three regions of Nigeria. In addition, this study highlights the need to establish sentinel surveillance sites for arboviruses in Nigeria.

## Results

### Sociodemographic characteristics and seropositivity of chikungunya and Zika arboviral infection in the study population

A total of 871 participants were recruited from three geographical regions for this study. Among these, 17.5% (152/871) were from Abia (Southern Nigeria), 34.4% (300/871) were from Kaduna (Northern Nigeria), and 48.1% (419/871) were from Nasarawa (Central Nigeria). The age of the participants ranged from 0 months to 80 years, with a mean age of 36.6 years.

**Fig. 1:**
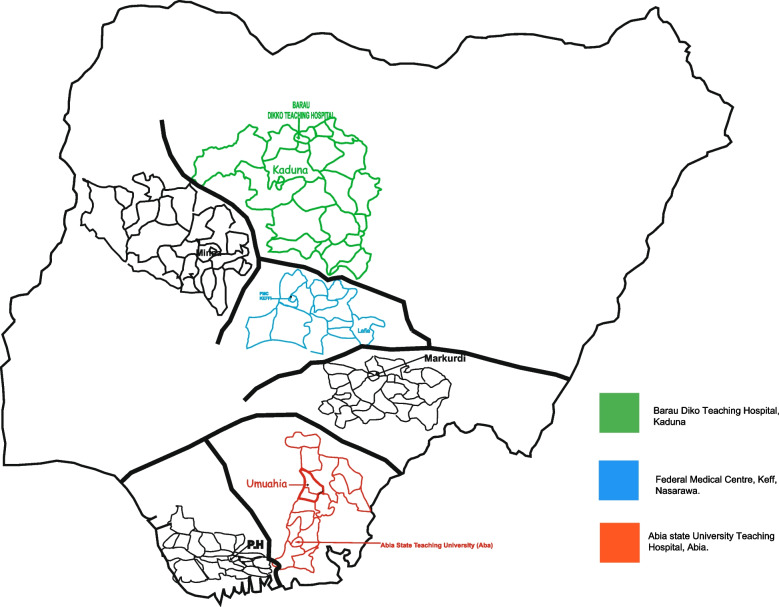
Chikungunya and Zika arboviral study sites in Nigeria

The study cohort overall IgG seropositivity for CHIKV was significantly higher [64.9% (565/871); 95% CI (0.61–68)] than for ZIKV [19.2% (172/871); 95% CI (0.19–0.20)], while the CHIKV-ZIKV antibody seropositivity was [6.2% (54/871); 95% CI (0.5–0.7)] (Fig. 1 & Table 1).

**Table 1:** Sociodemographic characteristics and seropositivity of chikungunya and Zika arboviral infection in the study population

**Region**	Chikungunya virus (CHIKV)						Zika (ZIKV)						Chikungunya-Zika cocirculation					
	Negative	Positive	Total examined	95% CI	OR	*P*	Negative	Positive	Total examined	95% CI	OR	*p*	Negative	Positive	Total examined	95%CI	OR	*p*
South	79 (52.0%)	73 (48.0%)	152		1	0.48	118 (77.6%)	34 (22.4%)	152		1	0.85	125 (82.2%)	27 (17.8%)	152		1	0.80
North	99 (33.0%)	201 (67.0%)	300	0.64–1.22	0.8		244 (81.3%)	56 (18.7%)	300	0.65–1.41	1.0		247 (82.3%)	53 (17.7%)	300	0.71–1.55	1.1	
Central	128 (30.5%)	291 (69.5%)	419	0.81–1.53	1.1		337 (80.4%)	82 (18.6%)	419	0.70–1.51	1.0		348 (83.1%)	71 (16.9%)	419	0.64–1.40	0.9	
**Sex**																		
Male	90 (35.7%)	162 (64.3%)	252		1	0.81	199 (79.0%)	53 (21.0%)	252		1	0.48	204 (80.9%)	48 (19.1%)	252		1	0.39
Female	216 (34.9%)	403 (65.1%)	619	0.71–1.31	1.0		500 (80.8%)	119 (19.2%)	619	0.79–1.64	1.1		516 (83.4%)	103 (16.6%)	619	0.80–1.72	1.2	
**Domicile**																		
Urban	197 (38.9%)	310 (61.1%)	507		1	0.06	409 (80.7%)	98 (19.3%)	507		1	0.99	422 (83.2%)	85 (16.8%)	507		1	0.50
Rural	78 (30.2%)	180 (69.8%)	258	0.41–1.02	0.6		206 (79.8%)	52 (20.2%)	258	0.56–1.75	1.0		209 (81.0%)	49 (19.0%)	258	0.67–2.24	1.2	
Slum	31 (29.2%)	75 (70.8%)	106	0.97–2.42	1.5		84 (79.3%)	22 (20.7%)	106	0.56–1.76	1.0		8 9(84.0%)	17 (16.0%)	106	0.44–1.49	0.8	
**Pregnancy status**																		
Pregnant	90 (39.1%)	140 (60.9%)	230		1	0.32	187 (81.3%)	43 (18.7%)	230		1	0.60	194 (84.4%)	36 (15.6%)	230		1	0.43
Nonpregnant	216 (33.7%)	425 (66.3%)	641	0.62–1.16	0.9		512 (79.9%)	129 (20.1%)	641	0.61–1.32	0.9		526 (82.1%)	115 (17.9%)	641	0.56–1.27	0.8	
**HIV status**																		
HIV Positive	234 (39.7%)	356 (60.3%)	590		1	0.00	534 (90.5%)	56 (9.5%)	590		1	0.00	545 (92.4%)	45 (7.6%)	590		1	0.00
HIV Negative	72 (25.6%)	209 (74.4%)	281	0.38–0.71	0.5		165 (58.7%)	116 (41.3%)	281	0.32–0.65	0.5		175 (62.3%)	106 (37.7%)	281	0.13–0.28	0.2	
**Marital Status**																		
Married	227 (34.9%)	424 (65.1%)	651		1	0.78	528 (81.1%)	123 (18.9%)	651		1	0.27	545 (83.7%)	106 (16.3%)	651		1	0.15
Single	79 (35.9%)	141 (64.1%)	220	0.76–1.44	1.0		171 (77.7%)	49 (22.3%)	220	0.55–1.18	1.0		175 (79.5%)	45 (20.5%)	220	0.51–1.11	0.8	
**Malaria Status**																		
Malaria Positive	84 (34.7%)	158 (65.3%)	242		1	0.00	196 (81.0%)	46 (19.0%)	242		1	0.00	202 (83.5%)	40 (16.5%)	242		1	0.69
Malaria Negative	222 (35.3%)	407 (64.7%)	629	3.65–7.37	5.1		503 (80.0%)	126 (20.0%)	629	2.89–5.93	4.1		518 (82.3%)	111 (17.7%)	629	0.62–1.37	0.9	
**Blood product source**																		
Outpatient serum	457 (60.1%)	304 (39.9%)	761		1	0.00	663 (87.1%)	98 (13.9%)	761		1	0.00	738 (97.0%)	23 (3.0%)	761		1	0.00
Blood Bank serum	25 (22.7%)	85 (77.3%)	110	0.12–0.31	0.1		41 (37.3%)	69 (62.7%)	110	0.05–0.13	0.1		79 (71.8%)	31 (28.2%)	110	0.04–0.14	0.0	
Total (N)	306 (35.1%)	565 (64.9%)	871	0.61–0.68			699 (80.2%)	172 (19.2%)	871	0.19–0.20			871 (93.8%)	54 (6.2%)	871	0.5–0.7		

### Regions

Subgroup analysis also revealed a considerably higher level of detectable antibodies against CHIKV in the central region (69.5%, 291/419) than that in the northern and southern regions. (Table 2). ZIKV (22.4%, 34/152) and CHIKV-ZIKV (17.8%, 27/152) seropositivity was notably higher in the southern region than in the northern and central regions (Table 1).

**Table 2:** Signs and symptoms presented by chikungunya or Zika monoinfected patients

Sign and symptoms	Mono-infection (% sign & symptoms)	
	Anti-chikungunya positive (*N* = 565)	Anti-Zika positive (*N* = 172)
Headaches	84.1% (475/565)	93.0% (160/172)
Exanthema	37.2% (210/565)	31.3% (54/172)
Fever	87.1% (492/565)	95.9% (165/172)
Abdominal pain	17.8% (101/565)	7% (12/172)
Diarrhoea	14.5% (82/565)	4.7% (8/172)
Myalgia	70.6% (399/565)	55.8% (96/172)
Vomiting	15.8% (89/565)	26.2% (45/172)
Generalised body pains	90.6% (512/565)	62.2% (107/172)
Arthralgia	61.1% (345/565)	69.8% (120/172)
Edema	10.4% (59/565)	0.0% (0/172)
Maculopapular	0.0% (0/565)	8.1% (14/172)
Retro-orbital pain	2.1% (12/565)	14.5% (25/172)
Nausea	0.9% (5/565)	7.0% (12/172)
Non-purulent conjunctivitis	0.4% (2/565)	2.9% (5/172)
Leukopenia	50.4% (285/565)	45.3% (78/172)

### Sex

The antibody seropositivity against CHIKV among female participants (65.1%, 403/619) was slightly higher than that in males, whereas ZIKV (21.0%, 53/252) and CHIKV-ZIKV (19.1%, 48/252) cocirculation antibody seropositivity was remarkably higher in male participants. However, the odds of CHIKV-ZIKV cocirculation were 1.2 times higher in the female group than in the male group (Fig. 1 & Table 1).

### Place of domicile

A high level of antibody seropositivity against CHIKV [OR = 1.5, *p* < 0.05]; 70.8% (75/106)] and ZIKV (20.7%, 22/106) was observed among slum dwellers, while marked seropositive co-circulating antibodies against CHIKV-ZIKV 919.0%, 49/258) were observed among rural dwellers. The odds of CHIKV were 1.5 times higher in the slum group than in the other two groups (Fig. 1 & Table 1).

### Pregnancy status

Considerable detectable antibodies against CHIKV (66.3%, 425/641), ZIKV (20.1%, 129/641), and CHIKV-ZIKV cocirculation (17.9%, 115/641) were observed in the non-pregnant group than in the pregnant group (Fig. 1 & Table 1).

### HIV status

HIV-negative individuals had significantly higher antibody seropositivity against CHIKV (74.4%, 209/281). Similarly, antibody seropositivity against ZIKV (41.3%, 116/281) and CHIKV-ZIKV (37.7%, 106/281) were remarkably higher in the HIV-negative participants. The results were considered statistically significant (*p* < 0.005) (Fig. 1 & Table 1).

### Marital status

In the present study, single (unmarried) participants had slightly higher antibody seropositivity against ZIKV (22.3% 49/220) and CHIKV-ZIKV (20.5% 45/220), whereas married individuals demonstrated considerable seropositive antibodies against CHIKV (65.1%, 424/651) (Fig. 1 & Table 1).

### Malaria status

Malaria-negative participants had a marginal seropositivity antibody against ZIKV and CHIKV-ZIKV cocirculation, whereas malaria-positive participants showed a slightly higher antibody seropositivity against CHIKV. The odds of CHIKV antibody seropositivity were 5.1 times higher in malaria-negative patients than in malaria-positive patients. The results were statistically significant (*p* < 0.005) (Fig. 1 & Table 1).

### Blood product source

**Fig. 2:**
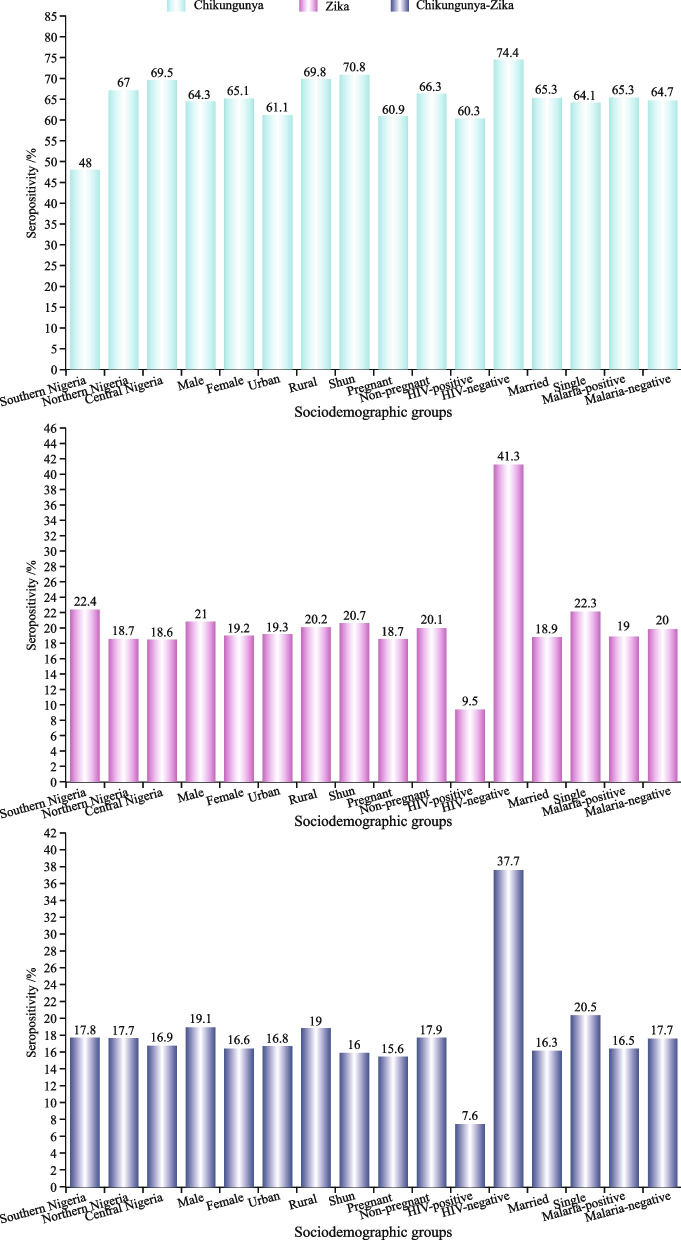
Sociodemographic characteristics and antibody seropisitivity of CHIKV, ZIKV and CHIKV-ZIKV cocirculation

Sera from the blood banks showed remarkable seropositivity against CHIKV (77.3%, 85/110)), ZIKV (62.7%, 69/110)) and CHIKV-ZIKV (28.2%, 31/110)) cocirculation compared to serum samples from outpatients. The results were statistically significant (*p* < 0.005) (Fig. 2 & Table 1).

### Age-specific seropositivity of Chikungunya, Zika, and Chikungunya-Zika cocirculation

**Fig. 3:**
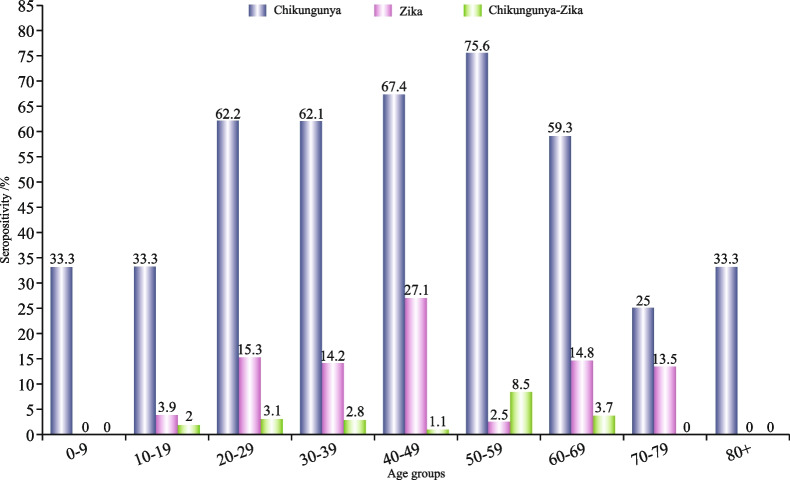
Age-specific seropositivity

The highest seropositivity of CHIKV antibody (75.6%, 62/82) was observed in the 50- to 59-year-old age group, and CHIKV-ZIKV-seropositive cocirculation antibodies (8.5%, 7/82) were observed in the same age group, whereas ZIKV-seropositive antibody (27.1%, 49/181) was observed in the 40- to 49-year-old age group. However, the results were not statistically significant (*p* < 0.05) (Fig. 3 & Table 3).

**Table 3:** Age specific seroprevalence of chikungunya, Zika and chikungunya-Zika cocirculation

Age (Years)	Chikungunya virus (CHIKV)			Zika virus (ZIKV)			Chikungunya-Zika coinfection		
	Negative	Positives	Total examined	Negative	Positives	Total examined	Negative	Positives	Total examined
0–9	2 (66.7%)	1 (33,3%)	3	3 (100%)	0 (0.0%)	3	3 (100%)	0 (0.0%)	3
10–19	34 (66.7%)	17 (33.3%)	51	49 (96.1%)	2 (3.9%)	51	50 (98.0%)	1 (2.0%)	51
20–29	74 (37.8%)	122 (62.2%)	196	166 (84.7%)	30 (15.3%)	196	190 (96.9%)	6 (3.1%)	196
30–39	120 (37.9%)	197 (62.1%)	317	272 (85.8%)	45 (14.2%)	317	308 (97.2%)	9 (2.8%)	317
40–49	59 (32.6%)	122 (67.4%)	181	132 (72.9%)	49 (27.1%)	181	179 (98.9%)	2 (1.1%)	181
50–59	20 (24.4%)	62 (75.6%)	82	80 (97.5%)	2 (2.5%)	82	75 (91.5%)	7 (8.5%)	82
60–69	11 (40.7%	16 (59.3%)	27	23 (85.2%)	4 (14.8%)	27	26 (96.3%)	1 (3.7%)	27)
70–79	6 (75.0%)	2 (25.0%)	8	7 (87.5%)	1 (13.5%)	8	8 (100%)	0 (0.0%)	8
80 +	4 (66.7%)	2 (33.3%)	6	6 (100%)	0 (0.0%)	6	6 (100%)	0 (0.0%)	6
Total(N)	306 (35.1%)	565 (64.9%)	871	699 (84.7%)	172 (19.8%)	871	720 (97.0%)	151 (17.3%)	871

## Discussion

In the present study, we employed sociodemographic parameters to investigate antibody seropositivity, endemicity, burden (high prevalence of a potentially serious disease), and multifarious spread of CHIKV, ZIKV and CHIKV-ZIKV-seropositive cocirculating antibodies in three regions of Nigeria. The overall rate of antibody seropositivity against CHIKV was 64.9%, while that against ZIKV was 19.6%. and CHIKV-ZIKV was 17.5%. In addition, 65.1% of CHIKV seropositive, 19.0% ZIKV seropositive, and 16.5% CHIKV-ZIKV seropositive patients were also positive for the malaria antigen. However, much lower seroprevalences were reported in different parts of Nigeria, most recently for CHIKV (29.3%, 25.1%) (Olawoyin and Kribs 2020; Vogels et al. 2019) and ZIKV (38.9%, 2.0%) (Masika et al. 2022; Rico-Mendoza et al. 2019; Zambrano et al. 2016). In the current study, we observed a 6.2% antibody seropositivity rate in CHIKV-ZIKV co-circulation patients. A cocirculation antibody seropositive rate of 7.6% (Asaga et al. 2022) was reported in the Columbia-Venezuela border and 12.0% (Norman et al. 2020) in South America. These varied results and findings can be explained by the cocirculation (Joseph et al. 2021; Edwards et al. 2016; Diallo et al. 2018; Zambrano et al. 2016) and common vector of transmission (*Aedes aegypti*) occurring in three geographic locations at the same time  (Asaga Mac et al. 2022; Ekong et al. 2022; Masika et al. 2022; Norman et al. 2020) . This result may also have been shaped by some level of arboviral antibody cross-reactivity from CHIKV (and other alphaviruses) and ZIKV (flaviviruses) past exposure immunity and arboviral vaccine (yellow fever). The serological assay used in this study was based on recombinant specific diagnostic IgG antigens derived from chikungunya and Zika. Thus, the antibodies were considered specific to CHIKV and ZIKV. The different levels of endemicity (seropositivity) for the two arboviruses in the three regions may be explained by different vector densities due to differences in vegetation, human population index, climate impact, vector adaptations, variations in temperature and humidity, flooding (which favors emergence and survival of arboviruses), changes in habitats and microclimates, and unplanned urbanization in the various regions that favor the transmission dynamics of mosquito-borne vectors (Zambrano et al. 2016; Omatola et al. 2020). The limited testing capacities of the regional health systems to accurately diagnose arboviral infections and distinguish them from other febrile illnesses explains the “hidden burden (as they remain usually undetected by the health services)” of this disease in various demographic groups. The sampling period (dry season through rainy season) also played a significant role in the current antibody seropositivity results across the three regions. Our findings are consistent with others, but much less extensive seroprevalence studies have been conducted in other parts of Nigeria, West Africa, and the rest of the world (Asaga Mac et al. 2022; Ekong et al. 2022; Masika et al. 2022; Norman et al. 2020; Ali et al. 2022; Carrillo-Hernández et al. 2018; Olawoyin and Kribs 2020; Otu et al. 2019; Vogels et al. 2019; Rothan et al. 2018; Omatola et al. 2020; Oluwole et al. 2022; Messina et al. 2016).

These two arboviral infections are more prevalent in older age groups (Asaga Mac et al. 2022; Gardner et al. 2018). This could be due to past infections (exposure over time), immunosenescence in old age, or long-standing immunity against arboviruses in older age groups and/or to increased vector exposure in relation to activities close to mosquito breeding habitats. Furthermore, older people maintain sedentary lifestyles because they sit for long periods in unscreened places, thus increasing their exposure to *Aedes* mosquito bites (day-feeding activity of *Aedes aegypti*).

In the present study, antibody seropositivity against CHIKV and ZIKV was remarkable in all three settlements (slum, rural and urban). This phenomenon may be due to rural–urban migration because of political conflict or fatigue, especially in northern and central Nigeria, travel and commercial activities, which may result in overcrowding, thereby driving unknown CHIKV and ZIKV antibody seroprevalences (Abdullahi et al. 2020; Bisanzio et al. 2018; Furuya-Kanamori et al. 2016). There is also the possibility of increased vector exposure near mosquito breeding habitats in urban areas and slums, such as refuse disposal or dump sites, unhygienic and poor sewage and drainage systems, and stagnant water in tires and tin containers, which act as suitable habitats for *Aedes* species (Asaga Mac et al. 2022; Norman et al. 2020; Ali et al. 2022; Carrillo-Hernández et al. 2018; Olawoyin and Kribs 2020; Otu et al. 2019; Iovine et al. 2017). Antibody seropositivity was particularly low in pregnant women with HIV infection. This could be explained by the strict adherence to antiretroviral medication and health-seeking behaviours among HIV-positive participants and the routine antenatal care during pregnancy because most of the study participants from the three regions were recruited from antiretroviral and antenatal units of tertiary healthcare centres.

The reasons for marked CHIKV seropositivity among malaria-positive and malaria-negative participants remain unclear. We do not know whether the presence of the malaria parasite reactivates or increases antibody seropositivity against CHIKV or whether CHIKV seropositivity increases or reactivates malaria infection (Oluwole et al. 2022; Perkins et al. 2016; Weaver and Lecuit 2015; Fauci and Morens 2016). Several studies have reported that concurrent or coinfection of malaria and arboviruses, especially in the tropics and subtropics, could increase seroprevalence rates (Norman et al. 2020; Furuya-Kanamori et al. 2016; Messina et al. 2016).

Serum samples from blood banks showed antibody seropositive against CHIKV, ZIKV and CHIKV-ZIKV cocirculating. This could be attributed to blood donation by asymptomatic individuals and the failure, lack and inability of regional health services or systems to diagnose and distinguish between malaria (most times, they screen for malaria but not arboviral infections) and other febrile illnesses.

## Limitations

The findings of our study are significant given that the transmission and multifarious spread of ZIKV (flaviviruses) and CHIKV (alphaviruses) are not well-documented in Nigeria. However, this study has several limitations. The cross-reactivity of IgG antibodies between flaviviruses and alphaviruses is well established and a confounding factor in serological studies investigating the seropositivity of arboviruses. All samples that tested positive for ZIKV, CHIKV or both were classified as positive for flavivirus or alphavirus, respectively. Due to the large sample size, it was impractical to conduct additional testing using techniques such as the plaque reduction neutralization test (PRNT) or other seroneutralization tests. In the present study, seropositivity for the two arboviruses was detected in the absence of antibodies against other flaviviruses or alphaviruses. Consequently, the antibody co-circulation of each of the two targeted arboviruses was confirmed.

The current study was tertiary hospital-based; thus, it did not accurately reflect the prevalence in the broader context of the Nigerian population. The COVID-19 pandemic posed a serious hindrance during sample collection, and many participants refused to enrol due to fear of the infection and the stigma associated with it. In addition, we did not perform plaque reduction neutralization tests (PRNT) and PCR to confirm CHIKV and ZIKV, which may have resulted in false-negative or false-positive results due to cross-reactivity (www.mikrogen.de, n.d.). Some of the antibody seropositive results may be due to arboviral vaccines or cross-reactivity with other arboviruses, such as dengue, West Nile virus, O'nyong'nyong virus, yellow fever virus, and Japanese encephalitis virus. Among the participants in the present study, there were more females than males, which may have led to bias and confounding other variables, as well as age.

## Conclusion

This study revealed the high seropositivity and endemicity of chikungunya, Zika, and chikungunya-Zika cocirculation antibodies in various Nigerian communities. The co-circulation of chikungunya and Zika antibodies is a chance occurrence that has sparked alarm in all tropical and subtropical regions around the world, not only because of the difficulty of making an accurate clinical diagnosis but also because of possible epidemiological complications. Acute febrile syndrome is a common feature of several arboviral infections in Nigeria, especially when causing indistinguishable febrile illnesses, and it is treated as a common infection, such as malaria and bacterial or fungal infections. Therefore, there is an increasing need to perform differential diagnosis in patients with acute febrile syndrome. This will assist clinicians and policymakers in designing and generating data, and implementing effective control measures.

## Method

### Study design and site

**Fig. 4:**
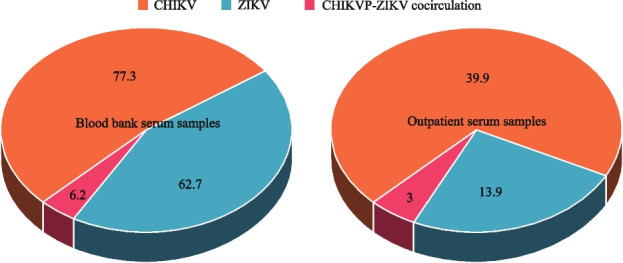
Blood product source seropositivity

This was a cross-sectional study conducted in three university teaching hospitals located in three geographical regions of Nigeria, namely, the Federal Medical Centre, Keffi, located in Nasarawa State, Central Nigeria; Abia State University Teaching Hospital, Aba, located in Southern Nigeria; and Baru-Diko Teaching Hospital, Kaduna, located in Northern Nigeria (Fig. 4) (Asaga Mac et al. 2022). It is estimated that more than 30 million people live in these regions. Approximately 45 percent of the population lives in urban areas, 40 percent in rural areas, and 15 percent in slums or informal settlements (Asaga Mac et al. 2022; Masika et al. 2022). In these areas, the average annual temperature ranges from 21 to 27°C, whereas in the interior lowlands, temperatures are generally above 27°C. The average annual precipitation level is 1,165.0 mm (Asaga Mac et al. 2022; Masika et al. 2022; Carrillo-Hernández et al. 2018; Olawoyin and Kribs 2020; Otu et al. 2019). In most parts of southern and central Nigeria, rainfall occurs throughout the year, with most rainfall occurring between April and October and minimal rainfall occurring between November and March in the north. The main occupations comprise formal and informal sectors, such as farming, trading, artisans, and career civil servants.

## Study population

The study population included all outpatients, pregnant women who enrolled for antenatal care and patients presenting with illness at the rapid-access healthcare and antiretroviral (people living with AIDS) units of the hospitals between December 2020 and November 2021 (Asaga Mac et al. 2022). These hospitals were purposefully selected to reflect diversity in terms of culture, religion, ethnicity, ecology, topographical and vegetation features, and different human activities. Inclusion criteria were all outpatients within an age range of 0 months to 80 years who agreed to participate in the study and signed the consent form, including children whose parents or guardians gave consent, while exclusion criteria were participants who were already undergoing treatment for malaria, those who refused to sign the consent form and seriously ill patients who were hospitalized (Asaga Mac et al. 2022).

A clinical research form (structured questionnaire) was used to obtain information that included questions on demographics, medical history, vital signs and symptoms, clinical evaluation, data on hospitalization, and a summary form. All study subjects were screened for malaria- and chikungunya-Zika-related symptoms (Table 1) (fever, headaches, rashes, joint pain, conjunctivitis and muscular pain). Detailed protocol information was made available and fully explained to the participants in English and their respective local languages before enrolment. The study participants signed an informed consent form after enrollment. Participants who could not read and write were asked to provide verbal consent and then to thumbprint, indicating that they were willing to participate in the study.

## Total number of samples collected

The sample size calculation (based on a 40% expected proportion of CHIKV and ZIKV infections in a total population of five hundred thousand patients with a confidence interval of 95% and accepted error of 5%), (https://select-statistics.co.uk/calculators/confidence-interval-calculator-population-mean/), showed a minimum sample size of 384 serum samples, which we increased to 871 (including those from the blood bank) samples to be able to analyse subgroups according to regions (Asaga Mac et al. 2022).

Venous blood (5 mL) was collected from each participant. Additionally, a local clinical diagnostic laboratory technician (located in the hospital who collected patient blood samples daily) collected 110 blood samples along with the clinical history from the blood banks of the three hospitals. We tested all serum samples at the study site for malaria parasites using a rapid antigen test kit (SD BIOLINE Malaria Differential P.f/Pan Ag RDT (HRP II + pLDH, Abbott, USA), according to the manufacturer's instructions. In summary, 5 µL blood sample was transferred into the sample well using the appropriate device included in the kit, and five drops of lysis buffer were added to the buffer well. The results were read visually after 15–20 min (Asaga Mac et al. 2022). The samples were then shipped on dry ice to the Institute of Virology in Freiburg, Germany, for molecular diagnostic analysis. The samples were stored at -20°C in preparation for testing for chikungunya and Zika antibodies.

## Laboratory analysis

For CHIKV and ZIKV, analyses were performed using the immunoblot assay recomLine Tropical Fever for the presence of the arboviral antibody serological marker IgG immunoblot (Mikrogen Diagnostik, Neuried, Germany) with ZIKV Nonstructural protein 1 (NS 1), ZIKV Equad (variant of the envelope protein with designated mutations to increase specificity), and CHIKV virus-like particle (VLP) according to the manufacturer’s instructions (Rodriguez-Morales AJ, et al 2016). The test is highly specific because of targeted mutations (specificity and sensitivity for CHIKV 100%; for ZIKV differentiation from other flaviviruses, 100% sensitivity and specificity for presumptive nonendemic areas, employing WHO-approved guidelines) (Rodriguez-Morales AJ, et al 2016). In summary, a test strip loaded with CHIKV and ZIKV antigens was incubated with diluted serum in a dish for 1 h. The cells were then washed three times. Peroxidase-conjugated anti-human antibodies (IgG-specific) were added and incubated for 45 min. The cells were then washed three times. A coloring solution was added after 8 min, and insoluble colored bands developed at the sites occupied by antibodies on the test strips (Rodriguez-Morales AJ, et al 2016).

## Arbovirus diagnostic serology interpretation

Due to mild and nonspecific symptoms, serological tests are essential for epidemiological investigations. However, these serological test interpretations may be hampered by notorious cross-reactive antibodies of flaviviruses and alphaviruses. Therefore, the interpretation of the test results in the present study may be reported as flavivirus seropositive for ZIKV and alphavirus seropositive for CHIKV.

## Statistical tests

Statistical analyses were performed using SPSS V. 28. Descriptive statistics were employed for the analysis of results, and we tested for associations between demographics and CHIKV and ZIKV antibody seropositivity, with the results deemed statistically significant at a *p* value ≤ 0.05, and odds ratios (OR) at a confidence interval (CI) of 95%.

## Ethics statement

The study protocol was reviewed and approved by the local ethics committee on human research at the Universitatsklinikum, Freiburg No. 140/19), and the local ethics committee on human research at the Tertiary Hospitals and National Ethics Committee on Human Research of Nigeria (No KF/REC/02/21).

## Supplementary Information

**Additional file 1.**

## Data Availability

All data are contained in the manuscript.

## Abbreviations

CHIKV: Chikungunya virus
CI: Confidence interval
VLP: Viral live particle
RDT: Rapid detection test
ZIKV: Zika virus
PRNT: Plaque reduction neutralization test
IgG: Immunoglobulin G
EQUAD: Zika mutant form of E protein
PCR: Polymerase Chain Reaction

## References

[CR1] Abdullahi IN, Akande AO, Muhammed Y, Rogo LD, Oderinde BS (2020). Prevalence Pattern of Chikungunya Virus Infection in Nigeria: A Four Decade Systematic Review and Meta-analysis. Pathogens and Global Health.

[CR2] Ali AA, Abdallah TM, Alshareef SA (2022). Maternal and perinatal outcomes during a Chikungunya outbreak in Kassala, eastern Sudan. Archives of Gynecology and Obstetrics.

[CR3] Asaga Mac P, Airiohuodion PE, Yako AB, Makpo JK, Kroeger A (2022). The Seroprevalence and Hidden Burden of Chikungunya Endemicity and Malaria Mono- and Coinfection in Nigeria. International Journal of Environmental Research and Public Health.

[CR4] Bisanzio D, Dzul-Manzanilla F, Gomez-Dantes H, Pavia-Ruz N, Hladish TJ, Lenhart A, et al. Spatio- temporal coherence of dengue, chikungunya and Zika outbreaks in Merida, Mexico. *PLoS Neglected Tropical Diseases*. 2018; 12: e0006298 10.1371/journal.pntd.0006298 10.1371/journal.pntd.0006298 PMC5870998 29543910

[CR5] Carrillo-Hernandez MY, Ruiz-Saenz J, Villamizar LJ, Gomez-Rangel SY, Martinez-Gutierrez M. Co-circulation and simultaneous coinfection of dengue, chikungunya, and Zika viruses in patients with febrile syndrome at the Colombian-Venezuelan border. *BMC Infectious Diseases*. 2018; 18: 61. 10.1186/s12879-018-2976-1 10.1186/s12879-018-2976-1 PMC5791178 29382300

[CR6] Carrillo-Hernández MY, Ruiz-Saenz J, Villamizar LJ, Gómez-Rangel SY (2018). Martínez-Gutierrez *M*Co-circulation and simultaneous coinfection of dengue, chikungunya, and zika viruses in patients with febrile syndrome at the Colombian-Venezuelan border. BMC Infectious Diseases.

[CR7] Coelho FC, Durovni B, Saraceni V, Lemos C, Codeco CT, Camargo S, de Carvalho LM, Bastos L, Arduini D, Villela DA, Armstrong M (2016). Higher incidence of Zika in adult women than adult men in Rio de Janeiro suggests a significant contribution of sexual transmission from men to women. International Journal of Infectious Diseases.

[CR8] Diawo Diallo, Ibrahima Dia, Cheikh T. Diagne, Alioune Gaye, Mawlouth Diallo, Chapter 4 - Emergences of chikungunya and Zika in Africa, Editor(s): Stephen Higgs, Dana L. Vanlandingham, Ann M. Powers, Chikungunya and Zika Viruses, Academic Press, 2018, Pages 87–133, 10.1016/B978-0-12-811865-8.00004-0.

[CR9] Edwards T, Signor L, Williams C, Donis E, Cuevas LE, Adams ER (2016). Coinfections with chikungunya and dengue viruses, Guatemala, 2015. Emerging Infectious Diseases.

[CR10] Ekong PS, Aworh MK, Grossi-Soyster EN, Wungak YS, Maurice NA, Altamirano J, Ekong MJ, Olugasa BO, Nwosuh CI, Shamaki D (2022). A Retrospective Study of the Seroprevalence of Dengue Virus and Chikungunya Virus Exposures in Nigeria, 2010–2018. Pathogens.

[CR11] Fauci AS, Morens DM (2016). Zika virus in the Americas—However, another arbovirus threat. New England Journal of Medicine.

[CR12] Fernandes-Matano L, Monroy-Muñoz IE, Pardavé-Alejandre HD, Uribe-Noguez LA, Hernández-Cueto MdlA, Rojas-Mendoza T, et al. Impact of the introduction of chikungunya and zika viruses on the incidence of dengue in endemic zones of Mexico. *PLoS Neglected Tropical Diseases*. 2021;15(12):e0009922. 10.1371/journal.pntd.0009922 10.1371/journal.pntd.0009922 PMC8638990 34855759

[CR13] Furuya-Kanamori L, Liang S, Milinovich G, Soares Magalhaes RJ, Clements ACA, Hu W, et al. Codis- tribution and coinfection of chikungunya and dengue viruses. *BMC Infectious Disease*. 2016; 16: 84. 10.1186/s12879-016-1417-2 PMID: 26936191 10.1186/s12879-016-1417-2 PMC4776349 26936191

[CR14] Gardner LM, Bóta A, Gangavarapu K, Kraemer MUG, Grubaugh ND. Inferring the risk factors behind the geographical spread and transmission of Zika in the Americas. *PLoS Neglected Tropical Diseases*. 2018; 12: e0006194. 10.1371/journal.pntd.0006194 10.1371/journal.pntd.0006194 PMC5790294 29346387

[CR15] Iovine NM, Lednicky J, Cherabuddi K, Crooke H, White SK, Loeb JC (2017). Coinfection with Zika and dengue-2 viruses in a traveler returning from Haiti, 2016: Clinical presentation and genetic analysis. Clinical Infectious Diseases.

[CR16] Joseph Ojonugwa Shaibu, Azuka Patrick Okwuraiwe *et al*. Sero-molecular prevalence of Zika virus among pregnant women attending some public hospitals in Lagos State, Nigeria. *European Journal of Medical and Health Science*. 2021 10.24018/ejmed.2021.3.5.1075.

[CR17] Masika, M.M.; Korhonen, E.M.; Smura, T.; Uusitalo, R.; Ogola, J.; Mwaengo, D.; Jääskeläinen, A.J.; Alburkat, H.; Gwon, Y.-D.; Evander,M.; et al. Serological evidence of exposure to Onyong-Nyong and chikungunya viruses in febrile patients of rural Taita-Taveta County and urban kibera informal settlement in Nairobi, Kenya. Viruses 2022, 14, 1286 10.3390/v14061286 10.3390/v14061286 PMC9230508 35746757

[CR18] Messina JP, Kraemer MU, Brady OJ, Pigott DM, Shearer FM, Weiss DJ, et al. Mapping global environ- mental suitability for Zika virus. *Elife*. 2016; 5: e15272. 10.7554/eLife.15272 10.7554/eLife.15272 PMC4889326 27090089

[CR19] Norman FF, Henríquez-Camacho C, Díaz-Menendez M, Chamorro S, Pou D, Molina I, Goikoetxea J, Rodríguez-Guardado A, Calabuig E, Crespillo C, Oliveira I, Pérez-Molina JA, López-Velez R; Redivi Study Group. Imported arbovirus infections in Spain, 2009–2018. *Emerging Infectious Disease*. 2020. 10.3201/eid2604.190443 PMC7101102 32186486

[CR20] Oluwole, T, Fowotade, A, Mirchandani,D Almeida,S. *et al*. Seroprevalence of some arboviruses among pregnant women in Ibadan, Southwestern, Nigeria, *International Journal of Infectious Diseases*, 116; 2022. 10.1016/j.ijid.2021.12.307.

[CR21] Olawoyin O, Kribs C (2020). Coinfection, Altered Vector Infectivity, and Antibody-Dependent Enhancement: The Dengue-Zika Interplay. Bulletin of Mathematical Biology.

[CR22] Omatola CA, Onoja BA, Fassan PK, Osaruyi SA, Iyeh M, Samuel MA, Haruna PU. Seroprevalence of chikungunya virus infection in five hospitals within Anyigba, Kogi State of Nigeria. Braz J Infect Dis. 2020;24(1):1–6. doi: 10.1016/j.bjid.2020.01.001. 10.1016/j.bjid.2020.01.001 PMC9392021 32001210

[CR23] Otu A, Ebenso B, Etokidem A, Chukwuekezie O (2019). Dengue fever - an update review and implications for Nigeria, and similar countries. African Health Sciences.

[CR24] Paniz-Mondolfi AE, Rodriguez-Morales AJ, Blohm G, Marquez M, Villamil-Gomez WE (2016). ChikDenMaZika Syndrome: The challenge of diagnosing arboviral infections in the midst of concurrent epidemics. Annals of Clinical Microbiology and Antimicrobials..

[CR25] Perkins TA, Siraj AS, Ruktanonchai CW, Kraemer MUG, Tatem AJ (2016). Model-based projections of Zika virus infections in childbearing women in the Americas. Nature Microbiology.

[CR26] Rico-Mendoza A, Porras-Ramírez A, Chang A, Encinales L, Lynch R. Cocirculation of dengue, chikungunya, and Zika viruses in Colombia. Rev Panam Salud Publica. 2019; 43:e49. 10.26633/RPSP.2019.49 10.26633/RPSP.2019.49 PMC6548069 31171921

[CR27] Rothan HA, Bidokhti MRM, Byrareddy SN (2018). Current concerns and perspectives on Zika virus coinfection with arboviruses and HIV. Journal of Autoimmunity.

[CR28] Rodriguez-Morales AJ, Villamil-Gómez WE, Franco-Paredes C. The arboviral burden of disease caused by cocirculation and coinfection of dengue, chikungunya and Zika in the Americas. *Travel Medicine and Infectious Disease*. 2016; 14: 177–179. 10.1016/j.tmaid.2016.05.004 10.1016/j.tmaid.2016.05.004 27224471

[CR29] Vogels CBF, Ru ¨ckert C, Cavany SM, Perkins TA, Ebel GD, Grubaugh ND (2019) Arbovirus coinfection and cotransmission: A neglected public health concern? *PLoS Biology* 17(1): e3000130. 10.1371/journal.pbio.3000130 10.1371/journal.pbio.3000130 PMC6358106 30668574

[CR30] Weaver SC, Lecuit M. Chikungunya virus and the global spread of a mosquito-borne disease. *New England Journal of Medicine*. 2015; 372: 1231–1239. 10.1056/NEJMra1406035 10.1056/NEJMra1406035 25806915

[CR31] www.mikrogen.de: (Supplementary file) https://www.mikrogen.de/produkte/produktuebersicht/testsystem/tropical-fever-igg.html.

[CR32] Zambrano H, Waggoner JJ, Almeida C, Rivera L, Benjamin JQ, Pinsky BA (2016). Zika virus and chikungunya virus coinfections: A series of three cases from a single center in Ecuador. American Journal of Tropical Medicine and Hygiene.

[CR33] Zambrano H, Waggoner JJ, Almeida C, Rivera L, Benjamin JQ, Pinsky BA (2016). Zika Virus and Chikungunya Virus CoInfections: A Series of Three Cases from a Single Center in Ecuador. American Journal of Tropical Medicine and Hygiene.

