# Supplementary figures and images for: Antibody seropositivity and endemicity of chikungunya and Zika viruses in Nigeria
Source: Anim Dis. 2023 Mar 23;3(1):7. doi: 10.1186/s44149-023-00070-2 (PMC10034229; doi:10.1186/s44149-023-00070-2)

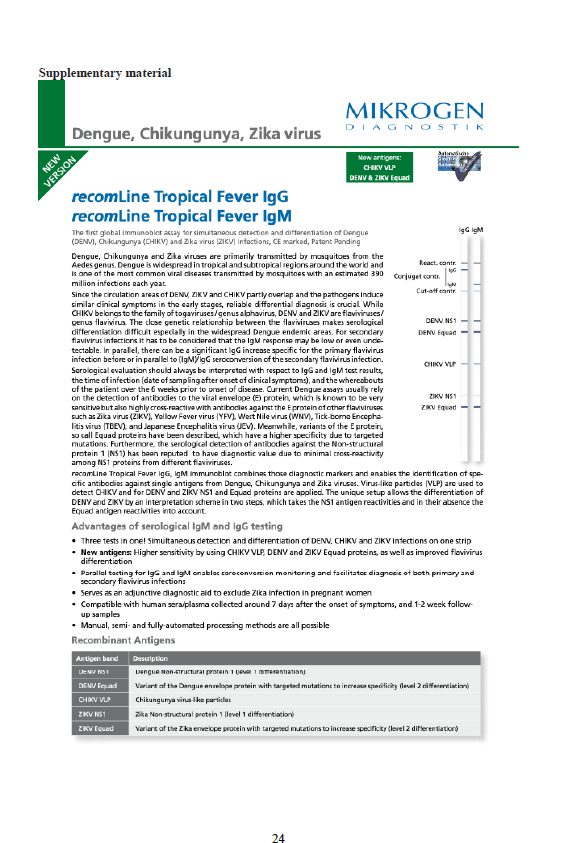

Supplement: Supplementary file 1 — Additional file 1. [file 44149_2023_70_MOESM1_ESM.png]
